# Impact of Surgical Intervention in Patients With Macrolide-Resistant *Mycobacterium avium* Complex Pulmonary Disease: A Multicentre Study

**DOI:** 10.1093/ejcts/ezag004

**Published:** 2026-01-06

**Authors:** Shota Nakamura, Fumie Kinoshita, Yukio Seki, Yohei Tsunoda, Yuta Hayashi, Taku Nakagawa, Kenji Ogawa, Toyofumi Fengshi Chen-Yoshikawa, Katsuo Yamada

**Affiliations:** Department of Thoracic Surgery, Nagoya University Graduate School of Medicine, Nagoya 466-8550, Japan; Department of Thoracic Surgery, Daido Hospital, Nagoya, 457-8511, Japan; Data Coordinating Center, Department of Advanced Medicine, Nagoya University Hospital, Nagoya, 466-8560, Japan; Department of Thoracic Surgery, NHO Nagoya Medical Center, Nagoya, 460-0001, Japan; Department of Respiratory Medicine, NHO Higashinagoya National Hospital, Nagoya, 465-8620, Japan; Department of Respiratory Medicine, NHO Higashinagoya National Hospital, Nagoya, 465-8620, Japan; Department of Respiratory Medicine, NHO Higashinagoya National Hospital, Nagoya, 465-8620, Japan; Department of Respiratory Medicine, NHO Higashinagoya National Hospital, Nagoya, 465-8620, Japan; Department of Thoracic Surgery, Nagoya University Graduate School of Medicine, Nagoya 466-8550, Japan; Department of Thoracic Surgery, Daido Hospital, Nagoya, 457-8511, Japan; Department of Thoracic Surgery, NHO Higashinagoya National Hospital, Nagoya, 465-8620, Japan

**Keywords:** *Mycobacterium avium* complex pulmonary disease, macrolide-resistant, clarithromycin, ethambutol, surgical intervention, drug resistance

## Abstract

**Objectives:**

Treatment for patients with macrolide-resistant *Mycobacterium avium* complex (MR-MAC) pulmonary disease is a major clinical challenge, as pharmacologic options are limited and outcomes with antibiotics alone are unsatisfactory. Although surgical intervention has been considered in selected cases, clinical evidence specific to MR-MAC is limited. This study aimed to compare the clinical outcomes of surgical intervention for MR-MAC pulmonary disease to those of non-resistant cases.

**Methods:**

This multicentre study included 248 patients who underwent pulmonary resection for MAC pulmonary disease. Among them, 34 patients (13.7%) had MR-MAC, which was defined as isolates with a clarithromycin minimum inhibitory concentration of ≥32 mg/L. Clinical outcomes were compared between the MR-MAC and non-MR-MAC groups. A multivariable analysis was conducted to identify risk factors for infectious relapse.

**Results:**

In the MR-MAC and non-MR-MAC groups, the 5-year overall survival, 5-year relapse-free survival and postoperative complication rates were 100% and 98.5%, 85.4% and 67.9%, and 8.8% and 11.9%, respectively (*P* = .72, .47, and .78, respectively). Multivariable analysis revealed older age and lack of amikacin use as independent risk factors for infectious relapse, but not macrolide resistance.

**Conclusions:**

Surgical resection is a viable and safe therapeutic option for selected patients with MR-MAC pulmonary disease, with long-term infection control comparable to that of non-MR-MAC cases. These findings support early surgical intervention in carefully selected patients with localized destructive lesions.

## Graphical Abstract

**Figure:**
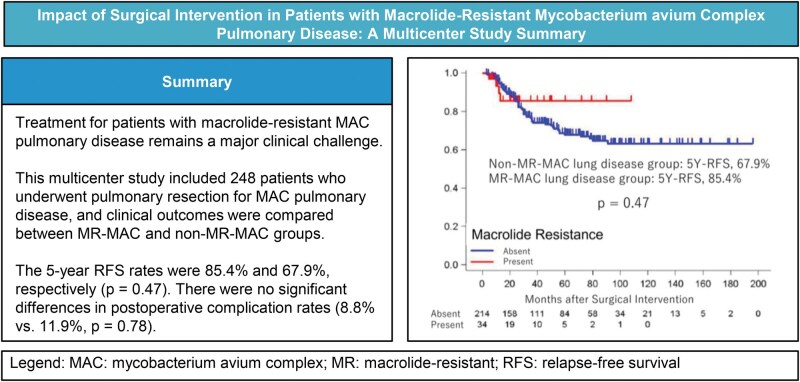

## Introduction

Newer macrolides are essential components of multidrug regimens for *Mycobacterium avium* complex (MAC) pulmonary disease.^1–4^ Current guidelines recommend a combination of a macrolide, ethambutol, and rifampicin.^1^^,^^2^^,^^5^ However, deviations from these recommendations, such as macrolide monotherapy or prolonged administration without ethambutol, have been associated with the development of macrolide-resistant MAC (MR-MAC), particularly in patients with chronic pulmonary disorders such as bronchiectasis.^6^^,^^7^

Real-world analyses have shown that these deviations from guideline-recommended regimens are common in clinical practice, presenting a significant challenge to effective disease management.^8–11^ When macrolide resistance develops, the efficacy of standard antibiotic therapy declines dramatically, resulting in higher rates of treatment failure, relapse, and mortality.^6^^,^^7^^,^^12–15^ According to a 2007 statement by the American Thoracic Society (ATS) and the Infectious Diseases Society of America (IDSA), clinical management of macrolide-resistant MAC (MR-MAC) pulmonary disease is especially difficult because resistance significantly reduces the efficacy of standard antimicrobial regimens.^1^ A small case series by Griffith et al.^6^ suggested that resection combined with aminoglycoside therapy could improve outcomes, but subsequent reports have been scarce and limited in sample size.

To address this knowledge gap, we conducted a multicentre observational study to compare the clinical outcomes of surgical treatment for macrolide-resistant *M avium* complex (MR-MAC) pulmonary disease to those for non-resistant cases, to determine whether surgical resection can achieve comparable long-term infection control despite macrolide resistance. To the best of our knowledge, this represents the largest cohort of surgically treated MR-MAC cases reported to date. Our findings aim to provide real-world evidence on the feasibility, safety, and efficacy of surgical management in this challenging patient population.

## Materials and methods

All studies involving human participants were performed in accordance with the ethical standards of the institutional and national research committees and with the 1964 Declaration of Helsinki, and its later amendments, or comparable ethical standards. This multicentre study was authorized by the central institutional review board of Nagoya University (Approval number: 2024–0326; Date of Approval: November 21, 2024). Informed consent was waived because of the retrospective design of the study.

### Study design

This multicentre retrospective observational study conducted in accordance with the Strengthening the Reporting of Observational Studies in Epidemiology (STROBE) guidelines, as well as the statistical and data reporting recommendations for the European Journal of Cardio-Thoracic Surgery.

### Study population

From April 2008 to March 2024, 289 consecutive patients underwent pulmonary resection for nontuberculous mycobacterial (NTM) pulmonary disease at 4 participating centres. Among them, 248 patients were confirmed to have MAC pulmonary disease based on the 2007 ATS/IDSA criteria and were included in this study.^1^

#### Inclusion criteria

1. Confirmed MAC pulmonary disease according to the 2007 ATS/IDSA diagnostic criteria.
2. Received ≥3 months of preoperative multidrug chemotherapy.
3. Localized disease deemed surgically resectable based on CT imaging.
4. Preserved cardiopulmonary function deemed adequate for lung resection (as determined by pulmonary function tests and exercise stress testing).

#### Exclusion criteria

1. Non-MAC NTM species
2. Diffuse or unresectable disease on preoperative imaging
3. Rapidly progressive disease during medical therapy, preventing surgical intervention
4. Required resection deemed excessive or predicted postoperative pulmonary reserve insufficient based on preoperative evaluation
5. Declined surgical treatment

### Surgical indications

**Figure 1.**
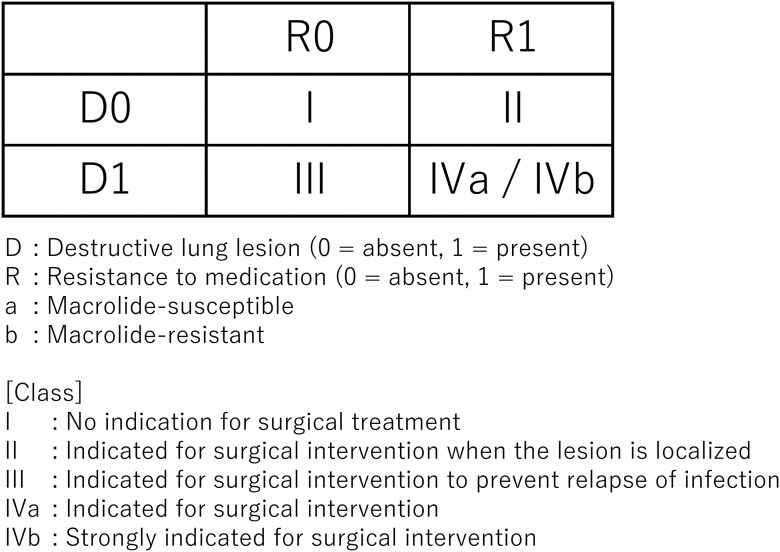
DR Classification for Surgical Indication in MAC Pulmonary Disease. Classification system based on the combination of destructive lesions (D) and resistance to medication (R) for determining surgical indications in MAC lung disease. Class II or higher was considered an indication for surgery, and class IV was defined as requiring surgical resection. MR-MAC was classified as class IVb. Reproduced with permission from Yamada K, et al. *Nihon Kokyuki Gakkai Zasshi*. 2021; 10(4):323–329. Copyright © 2021 The Japanese Respiratory Society. Abbreviations: DR classification, Destruction-Resistance classification; MAC, *Mycobacterium avium* complex; MR-MAC, macrolide-resistant *Mycobacterium avium* complex.

Surgical indications were determined based on the existence of 2 primary factors: (1) destructive airway lesions (eg, cavities and bronchiectasis) and (2) resistance to chemotherapy. A Destruction–Resistance (DR) classification system divided patients into Classes I–IV based on these 2 factors, with Class IV further subdivided into IVa and IVb to indicate macrolide resistance.^16^ Patients classified as Classes II, III, or IV were deemed eligible for surgical resection, with surgery deemed necessary in Class IV cases (**Figure 1**).

### Definition of macrolide resistance

Macrolide resistance was defined as a minimum inhibitory concentration (MIC) of ≥32 µg/mL. MIC determination was carried out using BrothMIC NTM (Kyokuto Pharmaceutical Industrial Co., Ltd) until 2022, then BrothMIC SGM was adopted in 2023 to improve measurement precision in accordance with updated guidelines.^17^

### Perioperative treatment

Chemotherapy regimens were tailored and administered according to ATS/ERS/ESCMID/IDSA guidelines.^2^ Oral multidrug regimens were administered daily, and if aminoglycosides were not initially included in the regimen, kanamycin (KM) or amikacin (AMK) was added preoperatively to improve antimicrobial efficacy. KM was given intramuscularly at 15 mg/kg, 2 to 3 times per week. For patients who had surgery before 2019, KM was given during the perioperative period and continued for up to 3 months postoperatively. For surgeries performed after 2019, intravenous AMK was administered for 8 weeks postoperatively.

All patients underwent at least 3 months of preoperative chemotherapy. Patients with rapidly progressive disease or extensive unresectable lesions, even after preoperative treatment, were not considered for surgery. Postoperative chemotherapy followed the same protocol as preoperative therapy. The duration of postoperative chemotherapy was determined by the resected tissue culture results: 2 years for culture-positive cases and 1 year for culture-negative cases. The combined preoperative and postoperative duration of aminoglycoside therapy ranged from 4 to 6 months, depending on the clinical outcome.

### Surgical procedures

The main goal of surgical resection was disease control. Surgery was used to treat irreversible lesions such as cavities and bronchiectasis. All surgeries were performed under general anaesthesia with the patient in the lateral decubitus position using one-lung ventilation, with a thoracoscopic approach being preferred whenever possible. Standard procedures were performed using a 3-port VATS technique, and when dense adhesions or complicated hilar anatomy were encountered, a posterolateral thoracotomy conversion was performed. Anatomical resections—segmentectomy or larger—were the standard procedure. Extensive pulmonary resection, defined as a procedure wherein 2 or more lesions were removed from separate lobes with at least one anatomical resection, was performed when disease involvement spread to multiple lobes, as previously described.^18^

### Definition of infectious relapse

Postoperative infectious relapse was defined as either (1) the isolation of MAC from sputum or bronchoscopy specimens or (2) the start of chemotherapy based on CT findings consistent with relapse, even in the absence of culture confirmation. The latter was confirmed by agreement of 2 independent respiratory physicians or radiologists.

### Data collection and study variables

The following variables were collected and recorded: age, sex, body mass index (BMI), Charlson Comorbidity Index (CCI), occurrence of respiratory symptoms, causative organism (Mycobacterium avium or Mycobacterium intracellulare), computed tomography (CT) findings (presence of destructive lesions), aminoglycoside use (KM, AMK, or none), time from initiation of medical therapy to surgery, surgical procedure (anatomical or non-anatomical), number of segments resected, surgical approach (minimally invasive or open thoracotomy), operative time, intraoperative blood loss, postoperative complications, and relapse status.

### Statistical analysis

Continuous variables were summarized as medians with ranges, and categorical variables as frequencies with percentages. The Wilcoxon rank-sum test was used for continuous variables and Fisher’s exact test for categorical variables when making group comparisons.

Overall survival (OS) was defined as the time from surgery to death for any reason, whereas relapse-free survival (RFS) was defined as the time from surgery to infectious relapse or death. Kaplan–Meier methods were used to determine survival distributions, and then compared using the log-rank test. For relapse-free survival (RFS) analysis, one patient with missing data on the time between chemotherapy initiation and surgery was excluded, leaving 247 evaluable patients.

The multivariate analysis was used to compare clinical outcomes in the MR-MAC and non-MR-MAC groups, as well as to determine whether macrolide resistance had an independent effect on long-term infection control (RFS). Covariates included age, sex, BMI, CCI, respiratory symptoms, causative organism, CT findings, aminoglycoside use, time to surgery, staged bilateral resection, number of resected segments, and surgical approach. Variables with a *P*-value <.2 in univariate analysis were included in the multivariate model to avoid excluding potential confounders. As a sensitivity analysis, we additionally performed an inverse probability of treatment weighting (IPTW) analysis based on the propensity score to account for potential baseline differences between the MR-MAC and non-MR-MAC groups. The propensity score was estimated using logistic regression with the same 12 covariates as previously described, and weighted Cox proportional hazards models were used to estimate the average treatment effect.

All statistical analyses were conducted using SAS version 9.4 (SAS Institute, Cary, North Carolina), with 2-sided *P* values <.05 considered statistically significant.

## Results

### Patient characteristics

Of the 289 patients who underwent surgical intervention for NTM pulmonary disease during the study period, 248 were diagnosed with MAC pulmonary disease and included in the analysis. Among them, 34 patients had MR-MAC, while the remaining 214 had non-MR-MAC (**Table 1**). Surgical procedures were performed based on indications defined by the DR classification (**Figure 1**). Anatomical resections were performed on 237 patients. A total of 44 patients (17.7%) underwent staged bilateral surgeries, with the surgeries being more frequent in the MR-MAC group compared to the non-MR-MAC group (38.2% vs 14.5%, *P *< .01). The median number of resected segments was 2.5 (range, 1.0–8.0), but significantly higher in the MR-MAC group (3.5 vs 2.5, *P *< .01). Simple lobectomy, segmentectomy, and wedge resection alone, and extended pulmonary resections were performed in 87, 56, 11, and 94 patients, respectively. None of the patients underwent a pneumonectomy (**Table S-2**). KM was administered perioperatively to 116 patients (46.8%), with 3 (8.8%) and 113 patients (52.8%) in the MR-MAC and non-MR-MAC groups, respectively. AMK was administered to 91 patients (36.7%), with 28 (82.4%) and 63 patients (29.4%) in the MR-MAC and non-MR-MAC groups, respectively. Seven of those treated with AMK received it via inhalation therapy using AMK liposome inhalation suspension (ALIS) rather than intravenous administration.

**Table 1.** Clinicopathological Characteristics of MR-MAC vs Non-MR-MAC Patients

Variable	Overall (*n* = 248)	MR-MAC (*n* = 34)	Non-MR-MAC (*n* = 214)	*P*-value
Median age, years (range)	59 (21-78)	65 (24-77)	58 (21-78)	<.01
Sex				.05
Male	55 (22.2%)	3 (8.8%)	52 (24.3%)	
Female	193 (77.8%)	31 (91.2%)	162 (75.7%)	
Body mass index (range)	19.6 (13.5-26.5)	19.4 (15.2-26.5)	19.7 (13.5-25.0)	.57
Preoperative symptoms, present	130 (52.4%)	17 (50.0%)	113 (52.8%)	.85
Pathogen				.83
*M. avium*	191 (77.0%)	27 (79.4%)	164 (76.6%)	
*M. intracellulare*	57 (23.0%)	7 (20.6%)	50 (23.4%)	
Charlson comorbidity index (≥2)	214 (86.3%)	30 (88.2%)	184 (86.0%)	1.00
Destructive lung lesions on CT, present	245 (98.8%)	34 (100%)	211 (98.6%)	1.00
Received aminoglycosides perioperatively				<.01
KM	116 (46.8%)	3 (8.8%)	113 (52.8%)	
AMK^a^	91 (36.7%)	28 (82.4%)	63 (29.4%)	
Not administered	41 (16.5%)	3 (8.8%)	38 (17.8%)	
Time from chemotherapy initiation to surgery, median months (range)	39 (2-1498)	80 (5-183)	35 (2-1498)	<.01
Staged bilateral lung resection	44 (17.7%)	13 (38.2%)	31 (14.5%)	<.01
Median resected number of pulmonary segments (range)	2.5 (1-8)	3.5 (2-8)	2.5 (1-7)	<.01
Minimally invasive approach	235 (94.8%)	33 (97.1%)	202 (94.4%)	1.00

a Includes 7 patients who received preoperative ALIS.

Abbreviations: AMK, amikacin; ALIS, amikacin liposome inhalation suspension; CT, computed tomography; KM, kanamycin; MR-MAC, macrolide-resistant *Mycobacterium avium* complex.

### DR classification distribution

Among the 248 included patients, DR classes II, III, IVa, and IVb were observed in 3 (1.2%), 22 (8.9%), 189 (76.2%), and 34 patients (13.7%) , respectively. All MR-MAC cases were classified as DR class IVb. When stratified by DR classification, there were no statistically significant differences in the OS or RFS of the 4 groups.

### Antibiotic regimens

The composition of multidrug therapy varied between the MR-MAC and non-MR-MAC groups. As shown in **Table S-1**, MR-MAC patients were more likely to receive fluoroquinolone-based regimens than clarithromycin-containing therapy. Perioperatively, in the non-MR-MAC and MR-MAC groups, KM was administered to 52.8% and 8.8% patients, whereas AMK was administered to 82.4% and 29.4% patients, respectively. These results demonstrated the temporal transition from KM- to AMK-based therapies.

Postoperative complications of grade ≥ 3 occurred in 22 patients (8.9%). The most common complication was a prolonged air leak, followed by empyema, postoperative bleeding, and pneumonia (*n *= 18, 2, 1, and 1, respectively). One treatment-related death occurred due to an acute exacerbation of interstitial pneumonia. One patient with prolonged air leak was treated with chemical pleurodesis, while 3 patients required reoperation. In terms of empyema, one patient underwent thoracoscopic debridement and pleural cavity lavage, and one patient required an open-window thoracostomy. All these patients recovered from the complication.

**Figure 2.**
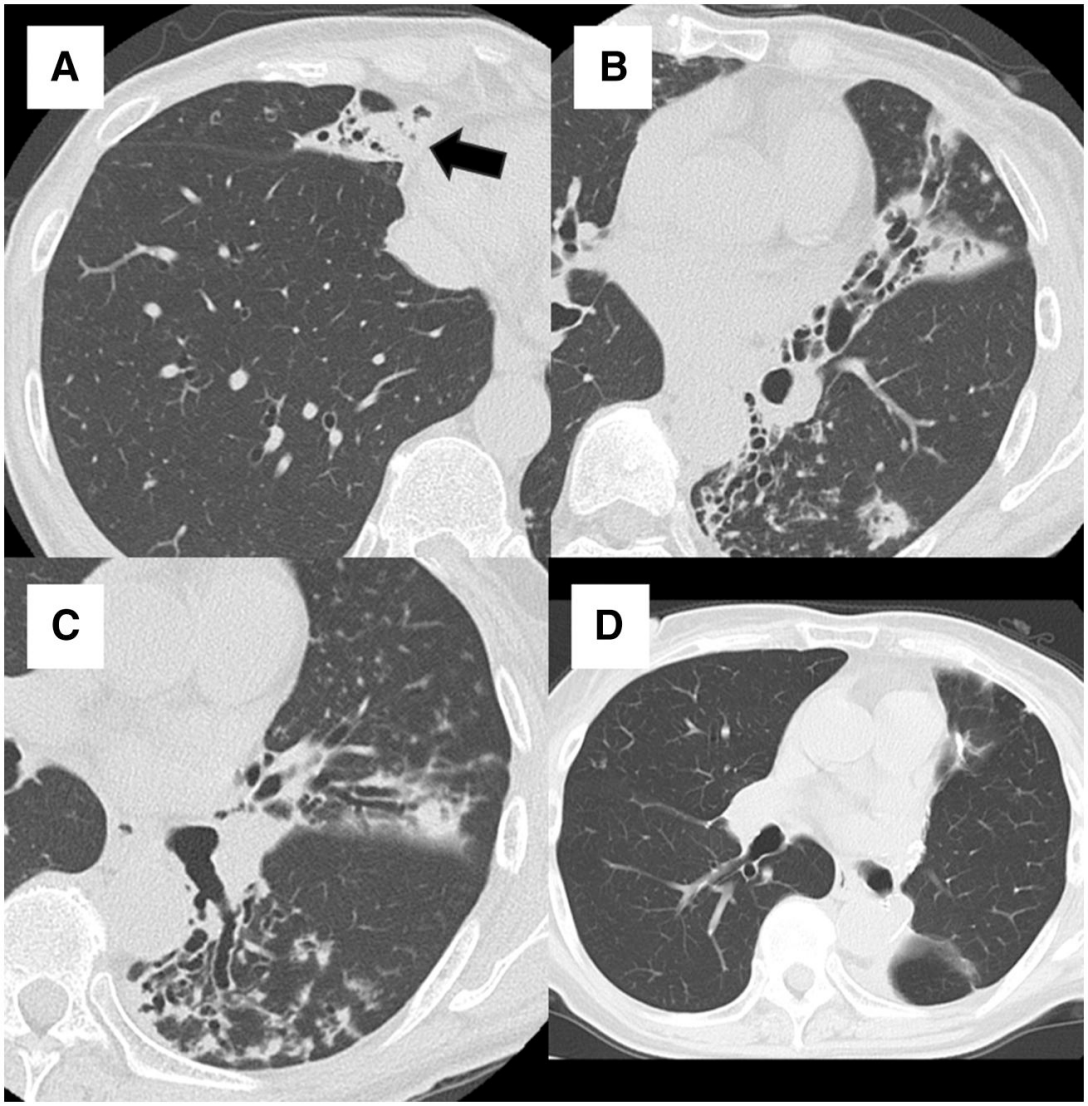
A Case of MR-MAC Pulmonary Disease. CT images of a 68-year-old woman with MR-MAC lung disease. (A-C) Preoperative axial views show destructive lesions in the right middle lobe, left lingula, and left lower lobe. Bilateral anatomical lung resections were performed in 2 stages. (D) Postoperative CT shows no residual lesions. The patient remains relapse-free and off antimicrobial therapy. Abbreviations: CT, computed tomography; MR-MAC, macrolide-resistant *Mycobacterium avium* complex.

**Figure 2** depicts a representative case of MR-MAC pulmonary disease. CT imaging revealed destructive lesions that correspond to Class IVb of the DR classification. The patient underwent 2-stage anatomical resections with no complications. Postoperative imaging revealed a successful resection, and the patient has been relapse-free and off antimicrobial therapy for 31 months.

### Overall survival and recurrence-free survival

The median follow-up period was 62.4 months. During this time, 189 patients (76.2%) were relapse-free, while 59 (23.8%) experienced an infectious relapse. Relapse was observed in 4 patients (11.8%) in the MR-MAC group and 55 (25.7%) in the non-MR-MAC group, with no statistically significant difference (*P *= .085). Among those with relapse, the median time from surgery to diagnosis was 21.0 months (range, 0–119 months): 3.0 months (range, 3–5 months) in the MR-MAC group and 26.0 months (range, 0–119 months) in the non-MR-MAC group (*P *= .002).

**Figure 3.**
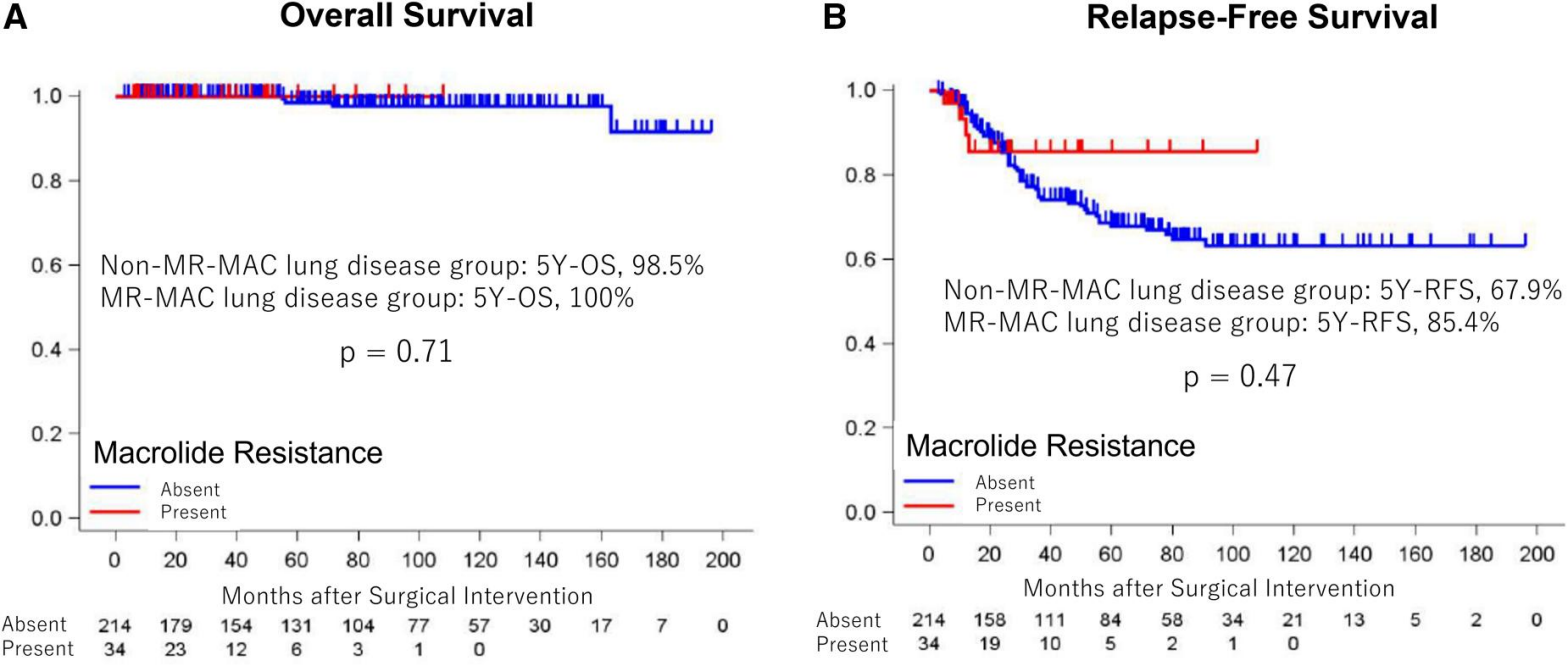
Survival Outcomes in MAC Pulmonary Disease. Kaplan–Meier curves comparing (A) OS and (B) RFS between patients with MR-MAC pulmonary disease and non-MR-MAC pulmonary disease. No significant differences were observed. Abbreviations: MR-MAC, macrolide-resistant *Mycobacterium avium* complex; OS, overall survival; RFS, relapse-free survival.

The MR-MAC and non-MR-MAC groups exhibited no significant differences in 5-year OS (5-year OS: 100% vs 98.5%, *P* = .7167; **Figure 3A**) or RFS (5-year RFS 85.37% vs 67.87%, *P* = .4695; **Figure 3B**).

Univariate and multivariate analyses for RFS are shown in **Tables 2 and 3**. Variables with a *P* value < .2 in univariate analysis—including age, preoperative aminoglycoside administration, duration of preoperative treatment, and CCI—were included in the multivariate model, as well as macrolide resistance status. The multivariate analysis revealed that older age and the absence of aminoglycoside administration were significant predictors of worse RFS. Macrolide resistance did not serve as an independent predictor of RFS. In the IPTW-weighted Cox model, macrolide resistance was not linked to worse RFS (hazard ratio = 0.67, 95% confidence interval 0.16–2.82, *P* = .582), which was consistent with the main analysis.

**Table 2.** Univariable Analysis for Relapse-Free Survival

Variables		*n*	HR	95% CI	*P*-value
MR- or non-MR-MAC lung disease					
	Non-MR-MAC	213	Ref.		
	MR-MAC	34	0.69	0.25-1.89	.47
Age		247	1.04	1.01-1.06	<.01
Sex					
	Female	192	Ref.		
	Male	55	1.38	0.80-2.36	.25
Body mass index		247	1.06	0.93-1.2	.37
Preoperative symptoms					
	Absent	118	Ref.		
	Present	129	0.88	0.53-1.45	.62
Pathogens					
	*M. avium*	190	Ref.		
	*M. intracellulare*	57	0.76	0.41-1.40	.38
Charlson comorbidity index					
	0-1	33	Ref.		
	≥2	214	0.65	0.34-1.24	.19
Destructive lung lesions on CT					
	Presence	244	Ref.		
	Absent	3	0.89	0.12-6.45	.91
Received aminoglycosides preoperatively					
	Not-done	41	Ref.		
	KM	115	0.87	0.48-1.57	.65
	AMK	91	0.25	0.09-0.68	<.01
Time from chemotherapy initiation to surgery		247	1	1.00-1.00	.08
Staged bilateral lung resection or single unilateral lung resection					
	Single unilateral	203	Ref.		
	Staged bilateral	44	1.29	0.65-2.54	.46
Minimally invasive approach or open approach					
	Minimally invasive	234	Ref.		
	Open approach	13	1.26	0.46-3.48	.65
Resected number of pulmonary segments		247	0.95	0.8-1.14	.61

Abbreviations: AMK, amikacin; CI, confidence interval; CT, computed tomography; HR, hazard ratio; KM, kanamycin; MR-MAC, macrolide-resistant *Mycobacterium avium* complex.

**Table 3.** Multivariable Analysis for Relapse-Free Survival

Variables		*n*	HR	95% CI	*P*-value
MR- or non- -MR-MAC lung disease					
	Non-MR-MAC	213	ref.		
	MR-MAC	34	0.82	0.28-2.37	.71
Age		247	1.04	1.02-1.07	<.01
Charlson comorbidity index					
	0-1	33	ref.		
	≥2	214	0.78	0.40-1.54	.48
Received aminoglycosides preoperatively					
	Not-done	41	ref.		
	KM	115	0.83	0.45-1.51	.54
	AMK	91	0.19	0.06-0.54	<.01
Time from chemotherapy initiation to surgery		247	1	1.00-1.01	.07

Abbreviations: AMK, amikacin; CI, confidence interval; HR, hazard ratio; KM, kanamycin; MR-MAC: macrolide-resistant *Mycobacterium avium* complex.

## Discussion

This multicentre study is the largest clinical evaluation of surgical intervention in patients with MR-MAC pulmonary disease with destructive lesions. Despite the challenges posed by drug resistance, surgical resection was performed safely and with high rates of long-term infection control, comparable to those seen in patients with macrolide-susceptible MAC. These findings confirm surgical intervention as a viable therapeutic option for MR-MAC pulmonary disease, expanding treatment options for this difficult-to-treat population.

Surgical resection for NTM pulmonary disease, including MAC infection, was used selectively. Ku et al.^11^ investigated the outcomes of 105 patients with NTM pulmonary disease, 41% of whom had MAC infection, and found a 26% postoperative complication rate and a 4.9% surgery-related mortality rate. Mitchell et al.^19^ carried out a retrospective study of anatomical lung resection in 236 NTM patients, approximately 80% with MAC. They reported a surgical mortality rate of 2.6%, a major complication rate (grade ≥III) of 11.7%, and a 4.2% incidence of bronchial fistula. These findings support the safety and efficacy of surgical resection and highlight the importance of a multidisciplinary approach. Given the limited efficacy of pharmacologic treatments for MR-MAC, surgical intervention has occasionally been considered in carefully selected patients, particularly those with bronchiectasis or localized NTM disease.^18^^,^^20–24^ Griffith et al.^6^ proposed combining surgical resection with aminoglycoside therapy for MR infections, a strategy supported by Morimoto et al.,^7^ who discovered that combination therapy outperformed either treatment alone. The 2007 ATS/IDSA guidelines also acknowledged the potential benefit of surgery in combination with chemotherapy for localized MAC pulmonary disease.^1^

Surgical resection is intended to remove areas of parenchymal destruction with a high mycobacterial burden, halting disease progression and lowering risks such as massive haemoptysis and mortality. Previous studies^18^^,^^21^^,^^24–26^ have shown that adjuvant pulmonary resection can improve outcomes in MAC pulmonary disease. A recent meta-analysis by Kim et al.^27^ supported this, demonstrating that adjunctive surgery significantly improves treatment success and microbiological cure rates. Notably, longer delays between medical therapy initiation and surgery have been associated with worse outcomes, supporting earlier intervention in suitable drug-resistant cases.^18^ The current study provides several clinically meaningful insights. First, despite the therapeutic challenge of macrolide resistance, MR-MAC patients had the same long-term RFS rate as non-MR-MAC patients. This finding suggested that, in patients with destructive airway lesions, the success of surgical intervention is primarily determined by the extent of resection rather than antimicrobial susceptibility. This finding was consistent with previous studies on non-MR-MAC disease, which have shown that antimicrobial penetration is limited in destroyed bronchiectatic areas and that surgical removal of these lesions improves outcomes.^16^^,^^27^ The current data extend this concept by demonstrating that a similar benefit can be obtained in MR-MAC disease. Second, the safety profile of surgical intervention was favourable, with a major complication rate of only 8.9% and no perioperative deaths in the MR-MAC cohort. This finding demonstrated that careful patient selection, multidisciplinary perioperative management, and the use of minimally invasive approaches can ensure surgical safety even in drug-resistant cases. These findings agree with those reported by Mitchell et al.^19^ Third, our findings support the therapeutic value of perioperative aminoglycoside therapy, which was found to be an independent predictor of lower relapse risk. This finding demonstrated the synergistic therapeutic effect of surgical resection and antimicrobial treatment in achieving long-term infection control, consistent with the findings of Morimoto et al.^7^ While this association may in part reflect the inherently stronger antimicrobial activity of amikacin compared with kanamycin, it is also important to consider potential time-period effects. In our cohort, the transition from KM to AMK occurred after 2019, during which surgical techniques and perioperative management continued to evolve. Thus, the improved outcomes observed in patients treated with AMK may reflect not only the drug’s intrinsic efficacy but also these contemporaneous advances in overall care. Taken together, these findings suggested that in patients with localized lesions and adequate pulmonary function, macrolide resistance alone should not rule out surgical resection.

Yamada et al.^16^ proposed a DR classification that takes into account the extent of pulmonary destruction and refractoriness to chemotherapy, providing a practical framework for guiding surgical decisions in MAC pulmonary disease. In our cohort, all patients met the criteria for Class II or higher, with many MR-MAC cases being Class IV, indicating advanced disease.

Although several studies have suggested that macrolide susceptibility might return after discontinuing macrolide therapy,^28^ the risk of rapid resistance recurrence upon re-exposure, as well as the difficulty of maintaining long-term disease control with antibiotics alone, continue to be major concerns. In contrast, our findings indicated that surgical resection provides favourable outcomes in patients with localized MR-MAC disease and should be considered a definitive treatment option in appropriate candidates.

Our study had some limitations. Its retrospective and observational design might introduce selection bias. Patients with MAC pulmonary disease were primarily managed by pulmonologists, and only those considered potential surgical candidates were discussed at multidisciplinary meetings with thoracic surgeons. As a result, the number of patients excluded due to rapidly progressive disease, unresectable lesions, or preoperative assessment was not consistently recorded across the participating centres. Despite these limitations, our results were consistent with the ATS/ERS/ESCMID/IDSA guidelines, which support surgical resection in selected patients, supporting the relevance of our findings.

## Conclusion

This study demonstrates that surgical resection, combined with appropriate perioperative antimicrobial therapy, including aminoglycosides, can achieve long-term disease control in patients with MR-MAC pulmonary disease, comparable to the outcomes in non-MR-MAC cases with localized, destructive lesions. The findings supported the importance of early surgical intervention in appropriately selected patients, regardless of macrolide resistance. Additional prospective studies are warranted to refine surgical indications and optimize multidisciplinary treatment strategies for this challenging patient population.

## Supplementary Material

ezag004_Supplementary_Data

## Data Availability

The datasets generated and/or analysed during this study are not publicly available due to privacy and ethical concerns. However, de-identified data may be obtained from the corresponding author upon reasonable request and as deemed appropriate.
