# Supplementary material for: Impact of Surgical Intervention in Patients With Macrolide-Resistant Mycobacterium avium Complex Pulmonary Disease: A Multicentre Study
Source: Eur J Cardiothorac Surg. 2026 Jan 6;68(1):ezag004. doi: 10.1093/ejcts/ezag004 (PMC12836428; doi:10.1093/ejcts/ezag004)
Supplement: ezag004_Supplementary_Data [file ezag004_supplementary_data.zip › supplimentallytables.docx]

Supplementary Table S-1. Preoperative antibiotic regimens in MR-MAC and non-MR-MAC groups

| Drug | MR-MAC (n = 34) | non-MR-MAC (n = 214) | *p*-value |
| --- | --- | --- | --- |
| Clarithromycin | 13 (38.2 %) | 187 (87.4 %) | < .01 |
| Azithromycin | 6 (17.6 %) | 15 (7.0 %) | 0.04 |
| Ethambutol | 25 (73.5 %) | 187 (87.4 %) | 0.06 |
| Rifampicin | 31 (91.2 %) | 183 (85.5 %) | 0.59 |
| Amikacin | 28 (82.4.0%) | 63 (29.4 %) | <.01 |
| Kanamycin | 3 (8.8%) | 113 (52.8%) | <.01 |
| Fluoroquinolone | 27 (79.4 %) | 57 (26.6%) | < .01 |
| Streptomycin | 0 (0%) | 6 (2.8 %) | 1.00 |
| Others | 0 (0%) | 3 (1.4 %) | 1.00 |

Supplementary Table S-2. Surgical procedures in MR-MAC and non-MR-MAC groups

| **procedures** | **Overall**  **(n = 248)** | **MR-MAC**  **(n = 34)** | **Non-MR-MAC**  **(n = 214)** |
| --- | --- | --- | --- |
| Anatomical resection | 237  (95.6%) | 34  (100%) | 203  (94.9%) |
| Not-extended resections | 154  (62.1%) | 13  (38.2%) | 141  (65.9%) |
| Lobectomy | 87 | 3 | 84 |
| Segmentectomy | 56 | 10 | 46 |
| Wedge resection | 11 | 0 | 11 |
| Extended resection | 94  (37.9%) | 21  (61.8%) | 73  (34.1%) |
| Bilobectomy | 18 | 3 | 15 |
| Bilobectomy + segmentectomy | 2 | 0 | 2 |
| Bilobectomy + segmentectomy + wedge resection | 1 | 1 | 0 |
| Lobectomy + bisegmentectomy | 6 | 0 | 6 |
| Lobectomy + segmentectomy | 24 | 5 | 19 |
| Lobectomy + segmentectomy + wedge resection | 4 | 1 | 3 |
| Lobectomy + wedge resection | 26 | 6 | 20 |
| Bisegmentectomy + wedge resection | 9 | 4 | 9 |
| Segementectomy + wedge resection | 3 | 0 | 3 |
| Segementectomy + wedge resection | 3 | 0 | 3 |
